# Recognizing Non-vertebral Manifestation of Mycobacterium avium Complex Osteomyelitis in a Patient With HIV

**DOI:** 10.7759/cureus.30199

**Published:** 2022-10-11

**Authors:** Dania A Shah, Melissa Kerkelis, Kara Asbury, Dana Sall

**Affiliations:** 1 Internal Medicine, HonorHealth, Scottsdale, USA; 2 Infectious Disease, HonorHealth, Scottsdale, USA

**Keywords:** opportunistic bacterial infection, bone and joint, disseminated mycobacterium avium complex disease, non-tuberculous mycobacterium, mycobacterium avium-complex

## Abstract

Disseminated *Mycobacterium avium* complex (MAC) infection is predominantly seen in immunocompromised individuals, such as those with HIV infection and CD4 counts <50 cells/mm3. It commonly manifests with nonspecific signs and symptoms, such as weight loss, fevers, night sweats, diarrhea, lymphadenopathy, hepatosplenomegaly, and cytopenias. This is a case of disseminated MAC osteomyelitis in an HIV patient. The lack of constitutional symptoms, in this case, presented a diagnostic challenge. In addition, nonvertebral osteomyelitis is an uncommon manifestation, making this case of disseminated MAC osteomyelitis a unique presentation.

## Introduction

*Mycobacterium avium* complex (MAC) is comprised of *Mycobacterium avium* and the closely related *Mycobacterium intracellulare*, both of which are ubiquitous in the environment. MAC is a frequent opportunistic infection seen in patients with HIV. Disseminated MAC is typically seen in advanced disease, and prophylaxis is generally initiated once CD4 counts fall below 50 cells/mm3. MAC infections have various clinical presentations in immunocompromised patients, including but not limited to, pulmonary disease, granulomatous hepatitis, lymphadenitis, colitis, brain abscess, osteomyelitis, skin and soft tissue abscesses, and more [1,2]. There may also be a lack of constitutional symptoms and negative blood cultures for MAC in disseminated MAC infection, leading to difficulty in early diagnosis [3]. It is essential to recognize MAC as a potential cause of osteomyelitis in patients with HIV, even if CD4 counts are above 50 cells/mm3. Timely diagnosis and initiation of appropriate treatment is crucial in management of MAC infections to prevent further dissemination.

## Case presentation

**Figure 1:**
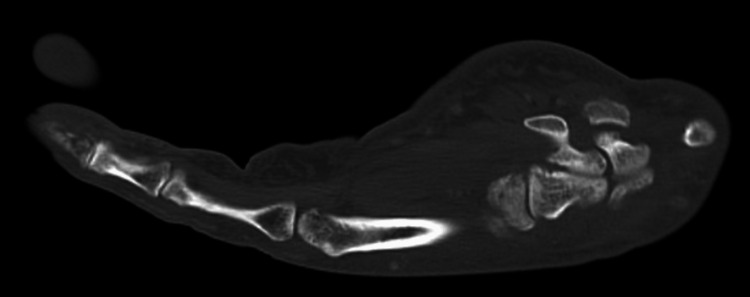
CT left-hand sagittal view with lytic appearance and cortical discontinuity of distal phalanx of index finger concerning for osteomyelitis.

This is a case of a 57-year-old man with a past medical history of recently diagnosed HIV, with an initial CD4 count of 90 cells/mm3, initiated on bictegravir/emtricitabine/tenofovir alafenamide, and prophylaxis with trimethoprim/sulfamethoxazole and acyclovir. Two months after the HIV diagnosis, the patient presented to the hospital with a left index finger infection. CT showed osteomyelitis of the distal phalanx of the index finger (Figure 1). Incision and drainage revealed frank purulent material under the deep fascia in the distal phalanx. Aerobic, anaerobic, fungal, and acid-fast bacilli (AFB) stains and cultures were obtained. However, the cultures remained negative at the time of discharge. During his hospitalization, the patient was treated with empiric vancomycin and piperacillin-tazobactam. He was discharged on empiric linezolid and levofloxacin, pending final cultures. 

**Figure 2:**
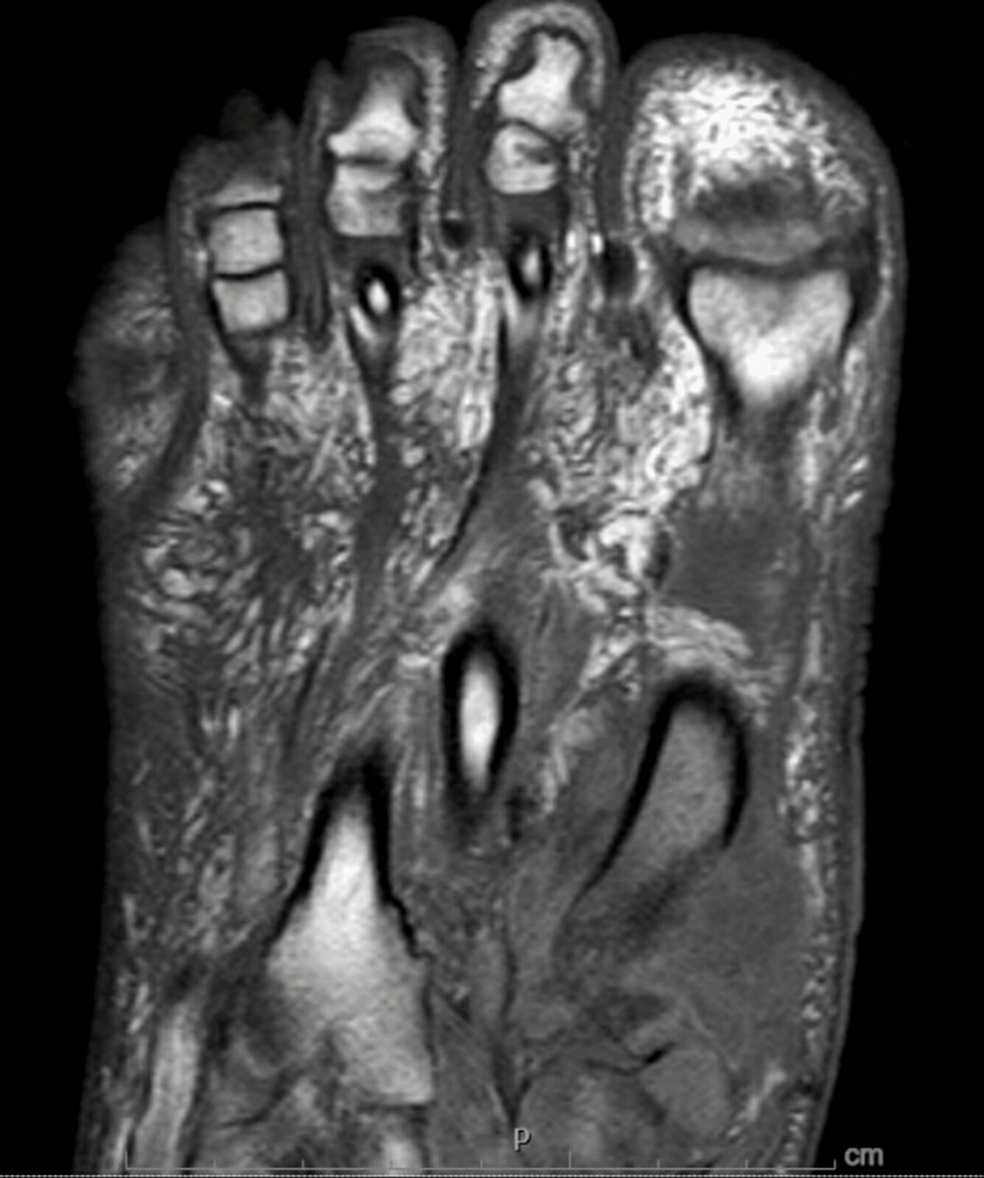
MRI right foot T1 coronal view with septic first tarsometatarsal joint with complex joint effusion, complex periarticular abscess measuring 35 mm, osteomyelitis of medial cuneiform and first metatarsal base with associated mildly displaced pathologic fracture of the first metatarsal base.

**Figure 3:**
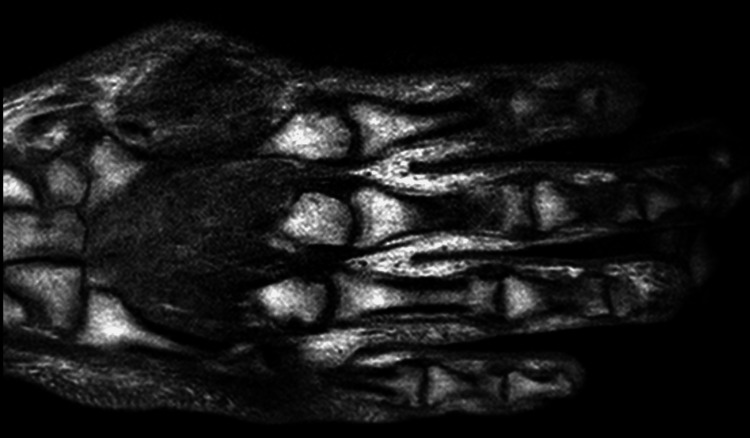
MRI left hand T1 coronal view with complex second through fourth carpometacarpal joint effusions with marked surrounding marrow edema, with moderate patchy low T1 marrow signal, suggestive of osteomyelitis and distal second phalanx osteomyelitis.

He subsequently presented to our hospital less than two months after the previous hospital discharge with right foot pain and swelling, along with left-hand swelling. The patient initially attributed the pain to trauma from dropping a heavy bottle on his right foot. Absolute CD4 count at the time of hospitalization was 181 cells/mm3, improved from 90 cells/mm3 at the time of antiretroviral therapy (ART) initiation. Quantitative HIV-1 RNA polymerase chain reaction (PCR) also showed a viral load of 31 copies/mL, improved from 608,000 copies/mL. MRI of the right foot was performed, demonstrating a septic first tarsometatarsal joint with complex periarticular abscess, along with osteomyelitis of the medial cuneiform and first metatarsal base (Figure 2). MRI of the left hand demonstrated unresolved distal second phalanx osteomyelitis as well as second to fourth carpometacarpal joint osteomyelitis (Figure 3). The patient underwent incision and drainage of abscess and excision of infected bone in the right foot. However, preliminary cultures were negative. 

Fortunately, upon review of the patient’s medical records, AFB cultures from the previous hospitalization grew MAC at approximately 7.5 weeks from the time cultures had been obtained. Thus, a preliminary diagnosis of disseminated MAC osteomyelitis was made, and the patient was initiated on azithromycin and ethambutol. Rifamycin antibiotics were not used due to drug-drug interaction with the patient’s ART. Intraoperative right metatarsal AFB culture ultimately grew MAC resistant to linezolid and moxifloxacin.

## Discussion

Disseminated MAC infection has been reported in patients with advanced immunosuppression caused by HIV, who are not on ART, or are not responding to ART therapy. While the localized infection is more often noted in patients on ART therapy [1]. MAC osteomyelitis is notoriously known to affect the vertebrae, frequently seen in patients with reconstituted CD4 counts following the initiation of ART therapy [4]. Disseminated nonvertebral MAC osteomyelitis of the foot and hand is an uncommon manifestation of this opportunistic pathogen. A previous literature review by Wood BR et al. [4] identified a total of 10 cases of nonvertebral MAC osteomyelitis in persons with HIV. On further literature review of PubMed with search terms “mycobacteria” or “mycobacterium”, “HIV”, and “osteomyelitis”, only a few additional case reports of nonvertebral MAC osteomyelitis were identified, highlighting the rarity of this manifestation [5,6,7].

The pathogenesis of spinal infection possibly involves immune activity in response to ART medication as part of the immune reconstitution syndrome (IRIS). On the contrary, studies have shown non-spinal infection to manifest in the setting of lower CD4+ count, secondary to unchecked mycobacterial replication and dissemination. We note that despite CD4+ count above 50 cells/mm3 and immediate introduction of ART therapy at the time of diagnosis, this patient developed non-spinal disseminated MAC infection in two joints. Additionally, this study underscores the underrecognition of MAC osteomyelitis in HIV and should remain high on differential diagnoses, despite adequate CD4+ count and ART therapy. To make a diagnosis, a bone biopsy should be obtained, and AFB stain and/or cultures should be performed. Treatment of MAC infection generally consists of a combination of a macrolide plus ethambutol. Azithromycin or clarithromycin are commonly used agents from the macrolide class [1]. Monotherapy with a single antimicrobial agent is avoided to decrease the risk of drug resistance, and susceptibility testing should be done on all isolates [8]. The duration of treatment is at least 12 months due to difficulty in its eradication [1].

## Conclusions

MAC osteomyelitis may be an underdiagnosed condition in patients with HIV who are on ART with adequate CD4 counts. Clinicians should maintain a high index of suspicion for MAC osteomyelitis when preliminary cultures are unrevealing and infection is refractory to treatment, despite adequate CD4 counts and appropriate ART therapy. As AFB cultures may take weeks for the result, it may be prudent to initiate treatment for MAC osteomyelitis if clinical suspicion is high. Early diagnosis and administration of treatment may help prevent further joint involvement and damage, as well as multiorgan involvement from disseminated disease.

## References

[1] (2022). Guidelines for the Prevention and Treatment of Opportunistic Infections in Adults and Adolescents with HIV. https://clinicalinfo.hiv.gov/en/guidelines/adult-and-adolescent-opportunistic-infection/mycobacterium-avium-complex-disease.

[2] Daley CL (2017). Mycobacterium avium complex disease. Microbiol Spectr.

[3] Gordin FM, Cohn DL, Sullam PM, Schoenfelder JR, Wynne BA, Horsburgh CR Jr (1997). Early manifestations of disseminated Mycobacterium avium complex disease: a prospective evaluation. J Infect Dis.

[4] Wood BR, Buitrago MO, Patel S, Hachey DH, Haneuse S, Harrington RD (2015). Mycobacterium avium complex osteomyelitis in persons with human immunodeficiency virus: case series and literature review. Open Forum Infect Dis.

[5] Xu X, Lao X, Zhang C (2019). Chronic Mycobacterium avium skin and soft tissue infection complicated with scalp osteomyelitis possibly secondary to anti-interferon-γ autoantibody formation. BMC Infect Dis.

[6] Gray ME, Liu PW, Wispelwey B (2018). Mycobacterium Avium complex vertebral osteomyelitis in the absence of HIV infection: a case report and review. BMC Infect Dis.

[7] Kawamura A, Sugawara H, Fukuchi T, Tanaka A (2021). Multidrug antibiotic therapy for a non-human immunodeficiency virus-infected patient with clarithromycin-resistant disseminated Mycobacterium avium complex disease. Cureus.

[8] Lin S, Hua W, Wang S (2022). In vitro assessment of 17 antimicrobial agents against clinical Mycobacterium avium complex isolates. BMC Microbiol.

